# AGING IN PLACE IN THE RURAL SOUTH

**DOI:** 10.1093/geroni/igad104.0430

**Published:** 2023-12-21

**Authors:** Caitlyn Nix

## Abstract

Aging in place in the rural south involves regionally unique concerns. Understanding the interaction between factors such as geographical isolation, smaller communities, and access to resources is critical for researchers, as results from research in urban areas may not generalize to this population. This symposium will outline regional barriers and protective factors related to aging in place from researchers in a distinctly rural area in the deep south. The symposium will begin with a discussion from Darby Mackenstadt on person-environment fit in a sample of older adults from Mississippi, with an emphasis on predictors of health status such as access to grocery stores and level of education. Carolyn Adams-Price will then review results from qualitative research on older adults’ self-reported attachment to home and local communities in rural Mississippi. Finally, Mary Dozier will give an overview of excessive household clutter as a barrier to maintaining functional independence for older adults enrolled in treatment studies for hoarding disorder in the state of Mississippi.

